# Implementing a Standard Operating Procedure Is Associated with Improved Vancomycin Target Attainment in Bone and Joint Infections: A Pre-Post Study

**DOI:** 10.3390/antibiotics14111087

**Published:** 2025-10-28

**Authors:** Moritz Diers, Juliane Beschauner, Maria Felsberg, Laura Isabell Kossack, Alexander Zeh, Karl-Stefan Delank, Natalia Gutteck, Felix Werneburg

**Affiliations:** 1 Department of Orthopedic and Trauma Surgery, Martin Luther University Halle-Wittenberg, Ernst-Grube Str. 40, 06120 Halle, Germanyjuliane.beschauner@uk-halle.de (J.B.);; 2 Department of Prosthodontics, Martin Luther University Halle-Wittenberg, Magdeburger Str. 16, 06108 Halle, Germany

**Keywords:** vancomycin, therapeutic drug monitoring, bone and joint infections, standard operating procedure

## Abstract

**Background**: Intravenous vancomycin is a mainstay for prosthetic joint infections, osteomyelitis, and implant-associated infections, yet real-world dosing frequently misses PK/PD targets. We assessed whether a ward-embedded standard operating procedure (SOP) improves target attainment and dosing efficiency. **Methods**: Single-centre, non-randomized pre-post study in an orthopedic service. SOP mandated weight-adapted loading dose, renal function-adjusted maintenance dosing, a 15–20 mg/L trough target, and scheduled TDM. Adults receiving ≥72 h IV vancomycin were included; major renal failure and incomplete TDM were excluded. Pre-SOP data were retrospective; post-SOP data were prospective (03/2024–06/2025). Primary outcome: proportion of troughs within 15–20 mg/L (first and repeated). Repeated measures were modeled with GEE. Time to first in-range trough used Kaplan–Meier (indexed by measurement number). **Results**: We included 154 patients (pre-SOP n = 58; post-SOP n = 96); baseline characteristics were broadly similar. Use of a weight-based loading dose rose from 31.0% pre-SOP to 100% post-SOP (*p* < 0.001). At the first trough, 17.2% vs. 26.0% were within 15–20 mg/L (*p* = 0.238). Across 847 troughs (pre = 319; post = 528), the in-range proportion increased from 28.2% to 41.7%, with subtherapeutic values declining from 38.2% to 26.3% and supratherapeutic values remaining nearly similar (33.5% → 32.0%). Time to first in-range trough shortened from a median of 4 to 2 measurements (log-rank *p* < 0.001). Post-SOP measurements had higher odds of being in range (aOR 1.68, 95% CI 1.29–2.20; *p* < 0.001), with marginal predicted probabilities of 33.4% (pre) vs. 47.8% (post). Dose adjustments per patient decreased from a mean 4.0 to 2.48 (*p* < 0.001). **Conclusions**: A pragmatic, orthopedic ward–embedded SOP for intravenous vancomycin improved pharmacologic precision: more measurements within target, fewer subtherapeutic exposures, faster target attainment, and fewer dose changes. These data support protocol-first implementation as an immediately actionable step toward more consistent vancomycin exposure in orthopedic care. Future work should integrate AUC-guided, model-informed precision dosing and evaluate clinical endpoints and generalizability across centres.

## 1. Introduction

Intravenous vancomycin plays a central role in the management of periprosthetic joint infections (PJI), osteomyelitis, and implant-associated complications—clinical scenarios that frequently require long-term antibiotic therapy. Therefore, PJI is one of the most feared complications in orthopedic surgery. Estimates from large observational studies suggest that about 1–2% of patients undergoing primary joint arthroplasty develop PJI, while the incidence may climb to around 10% in revision procedures [1,2]. Beyond being a leading cause of implant failure, PJIs are associated with severe consequences such as limb loss, mortality and substantial financial burden on healthcare systems and patients [3,4]. Their pathogenesis involves microbial adherence to implant surfaces via perioperative inoculation, hematogenous seeding or postoperative contamination; once established, bacteria form biofilms on prosthetic material, making eradication difficult and necessitating combined surgical debridement and prolonged antibiotic therapy.

Gram-positive organisms dominate the microbiology of PJIs. A large analysis of over 359,000 orthopedic inpatient encounters in the United States found that *Staphylococcus aureus* accounted for 48% of culture-confirmed surgical site infections and 1.13% overall, with 0.68% being surgical site and 0.28% bloodstream infections [5]. Methicillin-resistant *S. aureus* (MRSA) is particularly problematic: according to the U.S. Centers for Disease Control and Prevention, roughly 43% of hospital-associated *S. aureus* infections are MRSA, and European surveillance data show MRSA proportions ranging from <5% in northern Europe to >25% in parts of southern and eastern Europe [6]. Coagulase-negative staphylococci, such as *Staphylococcus epidermidis*, once dismissed as contaminants, are increasingly recognized as important pathogens for bone and joint infections due to their ability to form biofilms on implants.

Intravenous vancomycin remains a cornerstone for treating these Gram-positive infections. Its pharmacokinetic properties necessitate intravenous administration and dose individualization. Consensus guidelines for serious MRSA infections recommend a 400–600 mg·h/L area-under-the-concentration–time-curve (AUC) to minimum inhibitory concentration (MIC) ratio as the optimal pharmacodynamic target [7]. Initially, an AUC/MIC ≥ 400 was operationalized through trough concentrations of 15–20 mg/L; more recent evidence demonstrates that higher troughs increase the risk of vancomycin-associated acute kidney injury (VA-AKI) when daily AUC exceeds 650–1300 mg·h/L. Contemporary recommendations therefore emphasize early therapeutic drug monitoring (TDM), weight-based loading doses and renal function-adjusted maintenance dosing to achieve the AUC window while minimizing nephrotoxicity and other adverse effects. Despite clear guidelines, real-world dosing practices often fall short. In the cohort study by Haglund et al., involving 108 PJI patients, only 58.2% of 791 vancomycin trough measurements were within the 15–20 mg/L range; 18.5% were below and 23.4% were above, and 71% of patients required at least one dose adjustment [8].

Orthopedic departments often lack structured protocols, leaving decisions about loading doses, dose adjustment, and level timing to individual clinicians; this variability can result in under- or overexposure. To address this gap, our institution implemented a standard operating procedure (SOP) mandating weight-based loading, renal function–guided maintenance dosing, predefined trough targets, and scheduled TDM. While our earlier pre–post study [9] demonstrated a reduction in vancomycin-associated acute kidney injury under the SOP, it did not determine whether the protocol enhances pharmacologic precision. The present study therefore investigates the proportion of vancomycin trough concentrations within the therapeutic range before and after SOP implementation, characterizes the number and timing of dose adjustments, and explores predictors of successful target attainment in orthopedic inpatients. These process-focused endpoints complement the prior safety findings and delineate how protocolization translates into day-to-day dosing performance. The implemented SOP represents an antibiotic-stewardship–aligned dosing/TDM intervention aimed at standardizing dose selection, monitoring, and adjustments; it did not target antimicrobial selection, de-escalation, duration, or antibiotic consumption. It is not intended for chronic, indolent infection phenotypes, for which active antimicrobial therapy may be unnecessary after adequate source control; if antibiotics are indicated at all, targeted oral regimens—rather than intravenous vancomycin—might be preferred in these situations.

## 2. Results

### 2.1. Patient Characteristics

A total of 154 patients were included: 58 in the pre-SOP cohort and 96 in the post-SOP cohort. Baseline demographics and clinical indications were broadly comparable between groups (Table 1).

### 2.2. Dosing Implementation and First Trough

Use of a weight-based loading dose increased from 31.0% (18/58) pre-SOP to 100% (96/96) post-SOP (Fisher-exact text *p* < 0.001). At the first trough measurement, 17.2% of pre-SOP patients and 26.0% of post-SOP patients were within the 15–20 mg/L target range (Fisher-exact text *p* = 0.238).

### 2.3. Target Attainment Across All Measurements

**Figure 1:**
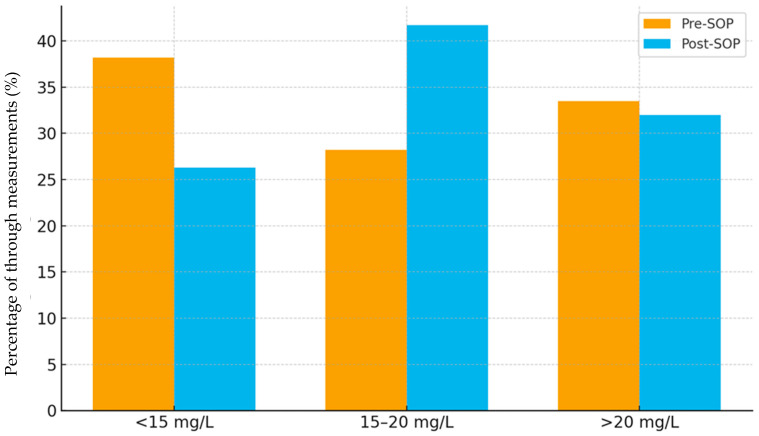
Distribution of vancomycin trough measurements by group (pre-SOP vs. post-SOP).

In total, 847 troughs were available (pre-SOP 319; post-SOP 528). Descriptively, the proportion of troughs within range increased from 28.2% pre-SOP to 41.7% post-SOP, with a concurrent decline in subtherapeutic values (38.2% → 26.3%) and a stable proportion of supratherapeutic values (33.5% → 32.0%) (Figure 1).

**Figure 2:**
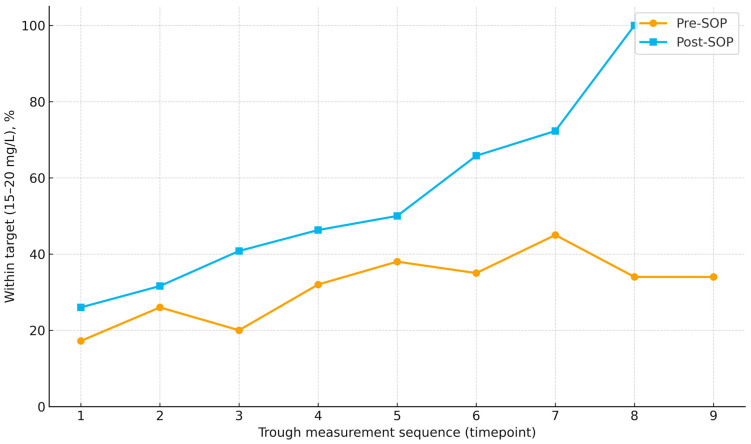
Proportion of vancomycin trough concentrations within the therapeutic range (15–20 mg/L) across successive measurements in pre-SOP and post-SOP cohorts. Points indicate the percentage within range at each time point; lines connect time points to illustrate trends. Number of patients decline at later time points (pre-SOP: 58 → 54 → 51 → 44 → 39 → 26 → 20 → 15 → 5; post-SOP: 96 → 89 → 85 → 68 → 63 → 47 → 26 → 9 → 0).

Across measurements, target attainment improved under the SOP, rising from 26.0% at the first assessment to 31.6%, 40.8%, 46.3%, and 50.0% at measurements 2–5, with a further increase to 65.8% at measurement 6; at measurement 7 the proportion reached 72.3%, and at measurement 8 all available troughs were within range (noting progressively smaller sample sizes at later time points). This pattern contrasts with the pre-SOP cohort, in which the proportions within range were generally lower and less consistently increasing (Figure 2).

**Figure 3:**
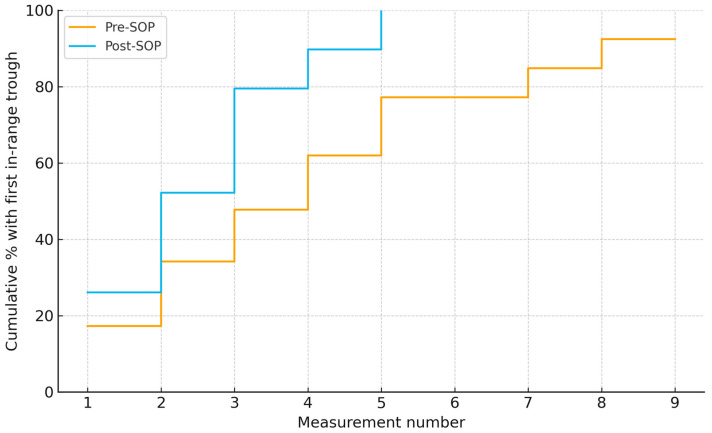
Time to first vancomycin trough within 15–20 mg/L by measurement number in pre- and post-SOP cohorts. Medians: 4 vs. 2 measurements (log-rank χ^2^ = 14.69, *p* < 0.001).

Using measurement number as the time index, time to first in-range trough was shorter post-SOP than pre-SOP median 2 vs. 4 (log-rank *p* < 0.001) (Figure 3).

### 2.4. Repeated-Measures Analysis

In the GEE model with patient-level clustering and the prespecified covariates, the post-SOP group had higher odds of being within the therapeutic trough range than the pre-SOP group (adjusted odds ratio 1.68, 95% CI 1.29–2.20, *p* < 0.001). The marginal predicted probability of being in range was 33.4% in the pre-SOP group versus 47.8% in the post-SOP group, an absolute difference of 14.4 percentage points. The group-by-measurement-number interaction was not significant (*p* = 0.11).

### 2.5. Dose Changes

Intravenous Vancomycin regimens required frequent modification in both cohorts. In the pre-SOP cohort (n = 58), 230 dose adjustments were recorded, corresponding to a mean of 4.0 adjustments per patient (SD 2.03; median 4; IQR 2.75; range 1–9). No patient completed therapy without modification. Patient-level frequencies were exactly one adjustment 8/58 (13.8%), two to three 16/58 (27.6%), four to five 22/58 (37.9%), and six or more 12/58 (20.7%).

In the post-SOP cohort (n = 96), 238 adjustments were documented overall, equating to a mean of 2.48 adjustments per patient (SD ≈ 1.35; median 2; IQR 1.5; range 1–6). Again, all patients had at least one modification. Frequencies were exactly one adjustment 25/96 (26.1%), two to three 50/96 (52.2%), four to five 17/96 (17.4%), and six or more 4/96 (4.3%).

**Figure 4:**
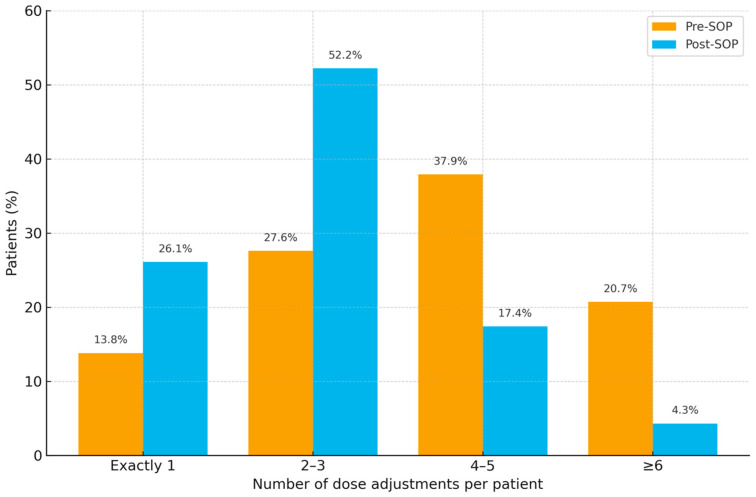
Distribution of vancomycin dose adjustments per patient in pre- and post-SOP cohorts across four categories (1, 2–3, 4–5, ≥6).

Figure 4 illustrates the distribution of dose adjustments per patient. After SOP implementation, patients were more likely to require only 1–3 adjustments (pre-SOP 24/58 [41.4%] vs. post-SOP 75/96 [78.1%]) and less likely to need ≥4 adjustments (pre-SOP 34/58 [58.6%] vs. post-SOP 21/96 [21.9%]). The reduction in dose adjustments per patient in the post-SOP group compared with the pre-SOP group was statistically significant (Mann–Whitney U = 1561; two-sided *p* < 0.001).

## 3. Discussion

### 3.1. Vancomycin PK and Target Attainment in Orthopedic Patients

Orthopedic inpatients—especially those with prosthetic joint infections (PJI) or other implant-associated infections—pose unique challenges for vancomycin therapy. Achieving adequate vancomycin concentrations in bone and joint tissues can be difficult due to pharmacokinetic variability and tissue penetration limits. In a recent cohort of PJI patients treated with IV vancomycin, *only about 58%* of measured troughs fell within the conventional 15–20 mg/L therapeutic range [8]. Approximately 18% of levels were sub-therapeutic and 23% were supratherapeutic despite dosing per guidelines. This indicates that many orthopedic patients are under- or over-exposed when using intravenous vancomycin. The same study reported that 71% of patients required at least one vancomycin dose adjustment during therapy, underscoring substantial inter-patient PK variability. Important patient factors influenced vancomycin levels: older age and impaired renal function correlated with higher troughs, suggesting a need for extra caution and individualized dosing in elderly or renally compromised orthopedic patients. On the other hand, local antibiotic strategies (e.g., vancomycin-loaded bone cement used in surgery) did not significantly affect systemic levels. Overall, these data highlight that standard weight-based dosing often fails to reliably hit PK/PD targets in PJI patients. It reinforces the rationale for closer TDM and possibly higher initial doses or AUC-guided approaches in orthopedic infections to ensure adequate drug exposure in bone. Indeed, achieving serum vancomycin troughs ≥15 mg/L has been associated with better infection control in PJI, although this must be balanced against nephrotoxicity risk [10]. Given the difficulty of consistently attaining targets in this population, there is also interest in alternative anti-staphylococcal agents that can overcome these PK challenges (discussed below).

### 3.2. Dose Adjustment Frequency, Time to Therapeutic Range, and Precision of Therapy

The need for frequent dose adjustments and the time required to reach therapeutic levels are practical markers of vancomycin dosing precision. Unfortunately, conventional vancomycin therapy often involves a protracted titration period. Studies in general hospital populations show that without aggressive management, it can take about 4–5 days for many patients to achieve target vancomycin levels [11]. Based on data from the Oxford University Hospitals (3767 patients receiving ≥24 h of IV vancomycin), initial dosing was similarly imprecise: only 26–32% of first vancomycin levels were within the target range, and only about 60% of patients on continued therapy achieved target troughs by day 5 [12]. This lag is clinically important—evidence suggests that delays in attaining therapeutic concentrations adversely affect outcomes [13]. Cardile et al. (2015) demonstrated that implementing a pharmacist-driven TDM program to expedite vancomycin titration (using higher initial doses and more frequent monitoring) significantly shortened time to target (median 3 days vs. 5 days) [11]. In this study patients who reached goal troughs <5 days had faster clinical improvement and were discharged earlier than those who reached targets later. Moreover, proactive dose-adjustment protocols halved the median hospital stay (7 vs. 14 days) and shortened time to stability (4 vs. 8 days) compared to a physician-guided approach. Crucially, the intensive TDM did not increase nephrotoxicity—AKI rates remained similar with or without the intervention. These findings underscore that more precise, responsive vancomycin dosing (with early adjustments) can improve patient outcomes in serious Gram-positive infections. Every 1–2 day reduction in time to therapeutic range matters, especially in deep orthopedic infections where timely bacterial clearance is critical. Thus, routine practice in many centers now is to make prompt dose modifications (often after the first trough) and to utilize tools that predict the needed adjustments more accurately (e.g., Bayesian calculators), rather than adhering to a “wait-and-see” approach over a week of suboptimal dosing.

### 3.3. Practical Considerations in Vancomycin TDM

Optimizing vancomycin therapy in practice involves several key TDM strategies: appropriate loading doses, timing of level collection based on renal function, and use of predictive dosing tools. Loading doses are widely recommended for severe infections—typically 25–30 mg/kg (actual body weight)—to rapidly attain therapeutic concentrations [14]. In critically ill or orthopedic patients with high bacterial burden, an initial loading dose can help achieve target trough/AUC within the first 24–48 h, rather than days later. Regarding level timing, standard practice is to obtain the first vancomycin level at (or near) steady-state, but this depends on the patient’s renal function and dosing interval. In patients on q12h dosing with normal renal function, a trough is usually drawn after 48 h [13]. If dosing is extended (q24h or longer for renal impairment), the first level is correspondingly timed earlier (e.g., before the 2nd dose with 24 h dosing) to avoid overshooting the target [13]. This renal function–based adjustment in TDM timing ensures that patients with slower clearance are assessed and adjusted promptly, rather than accumulating to toxic levels. Another practical aspect is the use of Bayesian forecasting and other model-informed precision dosing (MIPD) tools. These software-assisted methods can estimate a patient’s vancomycin AUC or future concentrations from limited early data, allowing clinicians to individualize doses without multiple trial-and-error level checks. Recent studies highlight the value of such tools: in one cohort, AUC-guided dosing via Bayesian software (with just a single concentration measurement) allowed earlier confirmation of target attainment and offered greater flexibility in sample timing [15]. Model-informed dosing not only improved safety—significantly reducing VA-AKI while maintaining efficacy—but also simplified the monitoring process. In practical terms, this means pharmacokinetic dose calculators can recommend dose adjustments immediately after the first level (even if taken early), helping hit the AUC goal by the second day of therapy. In summary, current best practices for vancomycin in orthopedic infections should involve: (1) front-loading the dose when needed, (2) timing drug levels intelligently based on the patient’s kinetics, and (3) leveraging PK/PD tools to guide dosing with precision. Together, these measures may make vancomycin therapy more predictable and effective, addressing many historical challenges of vancomycin TDM.

### 3.4. Potential Temporal Confounding and Secular Trends

As a single-centre, nonrandomized pre–post study, our findings may partly reflect secular trends unrelated to the SOP (e.g., staff turnover, laboratory turnaround times, or concurrent stewardship activities). During the study window, we are not aware of changes to the vancomycin assay, TDM platform, or institutional policies governing vancomycin selection, and no additional antimicrobial stewardship initiatives specifically targeting vancomycin were launched. The SOP roll-out included brief staff training that could improve practice independent of the protocol itself. Our GEE models adjusted for key case-mix variables, yet residual confounding cannot be excluded. The consistency of effects supports a protocol contribution; however, future interrupted time-series or stepped-wedge designs would better isolate causality.

### 3.5. How Our Findings Compare with—And Extend—The Literature

Our pre–post analysis shows that protocolization significantly enhanced *pharmacologic precision* of intravenous vancomycin in orthopedic inpatients.

First, the SOP drove universal uptake of weight-based loading (31.0% to 100%), a cornerstone step aligned with consensus guidance and supported by studies demonstrating faster early target exposure with loading in acute care settings [13].

Second, across all 847 troughs, the share within 15–20 mg/L rose from 28.2% pre-SOP to 41.7% post-SOP, while subtherapeutic values fell from 38.2% to 26.3%. This pattern mirrors the real-world challenge described by Gu et al. at Oxford: despite 84% compliance with a hospital dosing guideline, only 26% of first and 32% of subsequent levels were in range, and 55–63% of patients on continued therapy reached target by day 5 [12]. In that context, our post-SOP attainment rate across repeated measures—together with the clear downward shift in subtherapeutic exposure—suggests that a ward-embedded SOP can compress the oscillation between under- and over-dosing that typically plagues trough-guided practice.

Third, time-to-target improved substantially: using measurement number as an index, the median time to first in-range trough shortened from 4 to 2 measurements (*log-rank p* < 0.001). Few studies report attainment *as a trajectory*; where examined, delays of several days are common. Cardile et al. showed a pharmacist-driven TDM program shortened time to initial target from 5 to 3 days and improved downstream clinical efficiency, underscoring that each day gained matters for effective therapy [11]. Our analysis adds an orthopedic-ward perspective: a protocol that standardizes when to measure and how to adjust can halve the *measurement steps* needed to reach target, a pragmatic metric for surgeons and ward teams coordinating antibiotics around debridement and implant management.

Fourth, dosing *stability* improved: dose-change burden fell from a mean 4.0 to 2.48 adjustments per patient (*p* < 0.001), and the share needing ≥4 changes dropped from 58.6% to 21.9%. Haglund et al. reported frequent adjustments in a PJI-only cohort (71% required ≥1 change), despite guideline-concordant care [8]. While our absolute number of changes per patient differs—likely reflecting our broader indication mix and a deliberate, scheduled TDM cadence—the *direction* of effect is consistent across studies: standardization reduces reactive titration. Importantly, our adjusted repeated-measures model estimated 1.68-fold higher odds of being in range post-SOP (95% CI 1.29–2.20), with marginal predicted probabilities rising from 33.4% to 47.8%—evidence that the observed gains are not attributable to case-mix drift but to the protocol itself.

Finally, compared with orthopedic-specific cohorts, our results both corroborate and extend prior observations. Haglund et al. quantified the sheer difficulty of keeping troughs in the therapeutic window in PJI (58.2% in range; frequent adjustments; higher levels with older age and lower eGFR) [8]. Our data confirm the same patient-level gradients and demonstrate that a surgical-service-owned SOP can re-shape the exposure distribution: fewer lows, earlier on-target measurements, and a halving of per-patient titration steps across a broader orthopedic case mix. This extends earlier work from our center showing improved dosing control under the same SOP framework, now emphasizing target-attainment dynamics and dosing efficiency rather than adverse-event profiles. Together, these lines of evidence suggest that orthopedic units can realize substantial *therapeutic precision* gains with a pragmatic SOP today, while preparing for seamless adoption of AUC-guided dosing tomorrow. Notably, we did not systematically capture organism-level MICs for all targeted therapies and did not model AUC/MIC directly. Given MIC variability across testing methods and its clinical implications, future work should incorporate standardized MIC determination and AUC-guided, model-informed dosing to link exposure more directly to susceptibility and clinical outcomes.

## 4. Materials and Methods

### 4.1. Study Design and Setting

This single-centre, non-randomised pre–post study was conducted at the Department of Orthopedic Surgery of our university hospital. It assessed the impact of introducing a standardised vancomycin dosing and monitoring protocol on pharmacological target attainment and dosing adjustments. Data from patients treated before SOP implementation (pre-SOP) were retrospectively analysed, while data from patients treated after SOP implementation (post-SOP) were collected prospectively between 03/2024 and 06/2025.

### 4.2. Patient Selection

Patients were eligible for inclusion if they received intravenous vancomycin therapy during their inpatient stay at the orthopedic department, either as part of empiric treatment (typically ampicillin/sulbactam plus vancomycin) for presumed or culture-negative infections, or as targeted monotherapy with vancomycin in microbiologically confirmed cases. Patients were excluded if vancomycin was administered as a single dose only, if the duration of therapy was less than 72 h, or if severe renal dysfunction was present at baseline, defined as chronic kidney disease stage 4 or higher according to KDIGO criteria (eGFR < 30 mL/min/1.73 m^2^) or need for dialysis. Additional exclusion criteria included the concomitant use of nephrotoxic agents during vancomycin therapy (such as aminoglycosides or NSAIDs), incomplete documentation of vancomycin levels or renal function parameters, and antimicrobial therapy initiated or continued outside the orthopedic department.

### 4.3. Intervention: Standard Operating Procedure

In March 2024, an SOP for intravenous vancomycin therapy was implemented at the Department of Orthopedic Surgery at our university hospital. The SOP was developed to enhance both treatment safety and therapeutic precision by introducing standardized dosing practices and TDM. It was applied exclusively within the orthopedic service and followed institutional quality standards while aligning with internationally accepted guidelines for vancomycin use, including the revised consensus recommendations issued by ASHP, IDSA, PIDS, and SIDP [7].

Key elements of the SOP included the mandatory administration of a weight-based loading dose (25 mg/kg), renal function-adjusted maintenance dosing, a predefined target trough range of 15–20 mg/L, and clearly specified timing for TDM based on glomerular filtration rate (GFR). Trough levels were measured at steady state, defined as within 1 h prior to the respective dose depending on renal function:

- Before the 4th dose in patients with GFR ≥ 40 mL/min/1.73 m^2^;
- Before the 3rd dose in patients with GFR 20–39 mL/min/1.73 m^2^;
- 24 h after the loading dose in patients with GFR < 20 mL/min/1.73 m^2^ or receiving dialysis.

Dose adjustments were made using a structured matrix that integrated both trough level and renal function, with re-dosing intervals modified accordingly (Table 2). In patients with impaired renal function, re-dosing was guided by repeated TDM, with vancomycin administered only when serum levels dropped below 20 mg/L.

### 4.4. Data Collection

For each patient, we recorded demographic and baseline clinical information as well as details of vancomycin therapy. These included the indication for treatment, treatment rationale (empiric vs. targeted), the presence of a weight-based loading dose (yes/no), the start date of therapy, and, where available, the identified pathogen. Detailed dosing information—covering both the loading and maintenance doses—was documented. All vancomycin trough concentrations were captured, including the timing of the first trough measurement, the total number of trough measurements and their values. For each measurement, we noted whether a dose adjustment was made and, if so, the direction of the adjustment (increase or decrease). The total duration of vancomycin therapy (in days) was also recorded. Adverse events such as nephrotoxicity, ototoxicity and vancomycin flush syndrome were documented but not analyzed as outcomes in this present study.

### 4.5. Outcome Measures

The primary outcome was pharmacological target attainment, defined as the proportion of vancomycin trough concentrations within 15–20 mg/L. This was assessed for the first trough measurement (one per patient) and across all repeated measurements. Secondary outcomes were the proportion of patients receiving a weight-based loading dose and the number of dose adjustments per patient. Analyses of time to first in-range trough, direction of dose adjustments, overall mean trough levels, and covariate-specific associations were prespecified but treated as exploratory and are not reported in the main text.

### 4.6. Statistical Analysis

Data were analysed in IBM SPSS Statistics, version 27. Quantitative data were summarised in tables and figures. The primary outcome—being within the therapeutic vancomycin trough range (15–20 mg/L—was analysed across repeated measurements using generalized estimating equations (binomial family, logit link) with robust standard errors, clustering on patient ID and an exchangeable working correlation. Models were adjusted for prespecified covariates: group (post-SOP vs. pre-SOP), age, sex, baseline eGFR, loading dose (yes/no), treatment type (empiric vs. targeted), infection category, and measurement number. A group-by-measurement interaction was examined exploratorily. Results are reported as adjusted odds ratios with 95% confidence intervals and as marginal predicted probabilities. Time to first in-range trough (event = first trough 15–20 mg/L) was analysed using Kaplan–Meier curves with the log-rank test, indexing time by measurement number. Patients without an in-range trough were censored at their last available measurement. As a patient-level sensitivity analysis, we compared each patient’s proportion of in-range troughs between groups using the Mann–Whitney U test. The first trough and the use of a loading dose were compared with Fisher’s exact test; the number of dose adjustments per patient with the Mann–Whitney U test. Two-sided *p*-values < 0.05 were considered statistically significant.

### 4.7. Ethical Approval

For this retrospective, anonymized data analysis, the requirement for formal ethical approval was waived in accordance with institutional policy. A declaration of no objection was issued by the ethics committee of the Medical Faculty at Martin Luther University Halle-Wittenberg.

## 5. Conclusions

In this single-centre pre–post analysis, implementing a standardized vancomycin SOP in an orthopedic service measurably improved pharmacologic precision. Universal uptake of weight-based loading was achieved; the proportion of troughs within the 15–20 mg/L range increased and subtherapeutic exposure declined; time to first in-range trough was shortened from four to two measurements; and the per-patient dose-adjustment burden was markedly reduced. Adjusted repeated-measures modelling confirmed higher odds of target attainment post-SOP, supporting a true protocol effect rather than case-mix drift.

These findings provide pragmatic evidence that a ward-embedded, measurement-timed, and renal function–informed protocol can compress the usual oscillation between under- and over-dosing in orthopedic inpatients. Limitations include the single-centre, non-randomized design and the trough-based targets rather than AUC-guided dosing. Future work should evaluate AUC-guided, model-informed precision dosing within this framework and test generalizability in multicentre studies with clinical outcomes (infection control, nephrotoxicity, and length of stay). For now, a protocol-first approach offers an immediately actionable path to more timely, efficient, and consistent vancomycin exposure in orthopedic care.

## Figures and Tables

**Table 1:** Baseline demographic and clinical characteristics of orthopedic inpatients receiving intravenous vancomycin, stratified by study group (pre-SOP vs. post-SOP).

Characteristic	Pre-SOP	Post-SOP
Number of patients, n	58	96
Mean age, years (SD)	68.2 (11.0)	63.7 (13.1)
Male sex, n (%)	38 (65.5)	67 (69.8)
Clinical Indication for Vancomycin Therapy, n		
- Periprosthetic joint infection	33	58
- Septic arthritis	12	21
- Soft-tissue infection	8	13
- Spondylodiscitis	5	4
Type of therapy, n		
- Empiric	36	63
- Targeted	22	33
Pathogens identified (targeted therapy only), n		
- Staphylococcus epidermidis	12	22
- Staphylococcus aureus	7	9
- Staphylococcus capitis	3	2
Duration of vancomycin therapy, days (mean [SD])	8.6 (4.7)	9.3 (4.1)

**Table 2:** Vancomycin dosing adjustments based on trough level and renal function.

Trough Level (mg/L)	GFR (mL/min/1.73 m^2^) > 90	GFR 60–90 (mL/min/1.73 m^2^)	GFR 40–59 (mL/min/1.73 m^2^)	GFR 20–39 (mL/min/1.73 m^2^)	GFR < 20 (mL/min/1.73 m^2^)
<10	4 × 1 g	2 × 1.5 g	2 × 1 g	2 × 750 mg	TDM every 48 h; dose 1 g when through level < 20 mg/L
10–14.9	3 × 1.25 g	2 × 1.25 g	2 × 1 g	2 × 750 mg	
15–20	No dose adjustment required				
20.1–25	2 × 1.25 g	2 × 750 mg	2 × 500 mg	1 × 750 mg	
25.1–30	2 × 1 g	2 × 750 mg	2 × 500 mg	1 × 750 mg	
>30	Hold 24 h, recheck level				

Dosing recommendations were adapted according to estimated glomerular filtration rate (eGFR, mL/min) and measured vancomycin trough concentrations. For patients with severe renal impairment (eGFR < 20 mL/min), re-dosing was guided by repeated TDM every 48 h.

## Data Availability

The data presented in this study are available on request from the corresponding author. The data are not publicly available due to privacy and institutional restrictions.
